# On the concept of sloped motion for free-floating wave energy converters

**DOI:** 10.1098/rspa.2015.0238

**Published:** 2015-10-08

**Authors:** Grégory S. Payne, Rémy Pascal, Guillaume Vaillant

**Affiliations:** 1 Institute for Energy Systems, University of Edinburgh, Edinburgh EH9 3FB, UK; 2 Abengoa Seapower, ETTC, Alrick Building, The King's Buildings, Edinburgh EH9 3JL, UK; 3 Institut Jean Le Rond d'Alembert, Université Pierre et Marie Curie, Place Jussieu, Paris, France

**Keywords:** wave energy, boundary element method, WAMIT, optimization

## Abstract

A free-floating wave energy converter (WEC) concept whose power take-off (PTO) system reacts against water inertia is investigated herein. The main focus is the impact of inclining the PTO direction on the system performance. The study is based on a numerical model whose formulation is first derived in detail. Hydrodynamics coefficients are obtained using the linear boundary element method package WAMIT. Verification of the model is provided prior to its use for a PTO parametric study and a multi-objective optimization based on a multi-linear regression method. It is found that inclining the direction of the PTO at around 50° to the vertical is highly beneficial for the WEC performance in that it provides a high capture width ratio over a broad region of the wave period range.

## 1. Introduction

**Figure 1.**
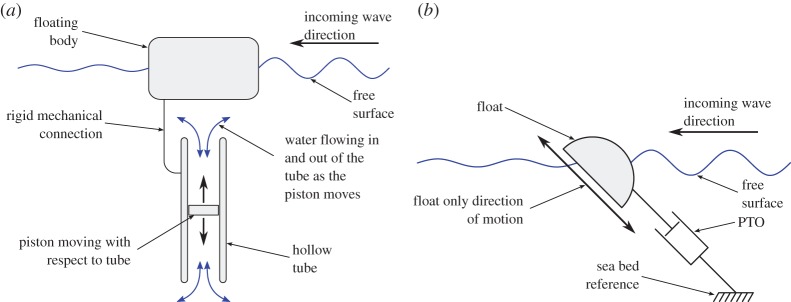
Section view schematic of a generic WEC reacting against water inertia (*a*) and side view schematic of the externally constrained sloped IPS buoy model tested by the University of Edinburgh (*b*).

Wave energy technology has now evolved from early academic research involving scaled model testings and theoretical investigations [1,2] to full-scale devices developed industrially and deployed in the ocean [3–5]. There is, however, no consensus on what type of wave energy converter (WEC) is the most promising and many WEC concepts are currently being investigated [6,7]. The main criteria for a WEC to be successful in the long term is to maximize the amount of energy harvested from the waves while minimizing cost and ensuring survivability. Although it is not necessary where it is the most exploitable [8], wave energy resource is generally more energetic in deep water environments than in shallow, coastal areas. However, increased water depth makes it more difficult and more costly to design WECs whose power take-off (PTO) mechanisms react against the sea bed. In such situations, an alternative approach is to use an inertial mass for the PTO to react against. This reduces significantly mooring loads as the mooring system is then only used for station keeping and not as a reference for the PTO. For such WECs to produce a significant amount of power, the mass reference needs to be large. If it is to be part of the device structure, it will be associated with significant costs. One way to alleviate this issue is to use water inertia as the PTO reference. One generic WEC concept that corresponds to this approach consists of a fully submerged tube, open at both ends and fitted with a piston able to slide inside. The tube is rigidly mounted onto a floating body and is thus subjected to wave-induced motions. The piston, constrained by the inertia of the water inside the tube, provides a reaction for the PTO mechanism (figure 1*a*). Energy is thus extracted from the relative motion between the piston and the tube. This concept was originally pioneered by the Swedish company Inter Project Services AB whose device (the IPS buoy) was fitted with a vertical PTO tube as in figure 1*a* [9].

In the 1990s, the University of Edinburgh adapted the IPS buoy by tilting the axis of the PTO tube away from vertical in an attempt to use both heave and surge wave induced motions of the water particles. In a first scaled model experimental investigation, the motion of the float was externally constrained to a single degree of freedom which was a translation along a sloped direction as shown in figure 1*b*. The PTO was referenced to the sea bed. More information on this study can be found in [10,11].

**Figure 2.**
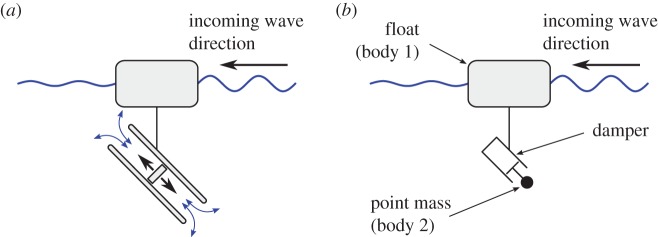
Section view schematics of a free-floating WEC reacting against water inertia (*a*) and against a point mass (*b*).

In a subsequent stage, a free-floating configuration was investigated. The main focus was to compare numerical modelling results with tank testing measurements [12,13]. In this configuration, the PTO was reacting against water inertia as depicted in figure 2*a*.

This paper presents a parametric optimization of a generic ‘sloped’ WEC whose PTO is reacting against an inertial reference. The behaviour of the system is modelled using the boundary element method (BEM) package WAMIT. The configuration studied is a simplification of the system shown in figure 2*a*. The PTO tube and piston system are replaced by a point mass connected to the float by a damper as shown in figure 2*b*. This simplification neglects the hydrodynamic force on the PTO tube and the hydrodynamic interactions between the piston and the fluid domain, but in return it alleviates the computational burden. It is anticipated that the configuration described in figure 2*a* could behave slightly differently from the model with a point mass, but it is believed that the overall dynamic of the simplified system is similar enough to reliably investigate the concept of slope.

The paper first provides a detailed derivation of the numerical model which is then verified against numerical benchmark data. A parametric study and a multi-objective optimization are then carried out using a multi-linear regression method.

## 2. Numerical model derivation

The BEM package WAMIT was originally used in the offshore industry [14–17] but has more recently been used to model the behaviour of WECs [12,13,18–21].

The WEC described in figure 2*b* is modelled as a two-body system: the float (body 1) and the point mass (body 2). Only the former is subjected to hydrodynamic and hydrostatic loads. These are computed using WAMIT. The equations of motion of the system are expressed in the frequency domain using an Eulerian approach. The motion amplitudes of both bodies are considered small with respect to the cross-sectional dimensions of body 1 and with respect to wavelength. Equations of motion are linearized thus neglecting the terms of order higher than 1.

### (a) System description and notations

The system is assumed to have a vertical plane of symmetry which is parallel to the direction of propagation of the waves. Consequently, motions of the system will be restricted to this plane. The only degrees of freedom considered are therefore surge, heave and pitch. Consequently, all forces and displacement components perpendicular to that plane are considered null. This also applies to all rotations and moment components whose axis are not perpendicular to that plane.

**Figure 3.**
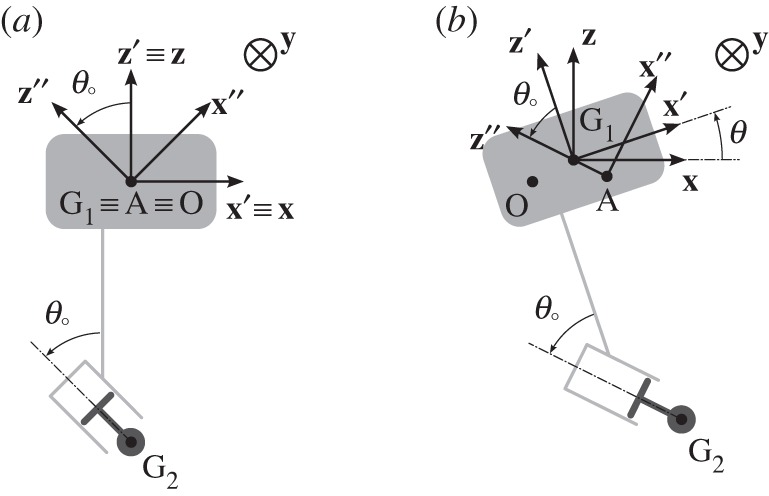
Schematic of the two-body system with coordinate systems and notations. (*a*) The system is at rest, whereas in (*b*) it is in motion, away from its equilibrium position. The symbol ⨂ represents the vector **y** being perpendicular to the plane of the sketch and pointing ‘into the page, away from the reader’.

The coordinate systems and the notations used are summarized in figure 3 in which the float (body 1) is shown in light grey and the point mass (body 2) is shown in darker grey.

$R_{0}=(O,\text{x},\text{y},\text{z})$ is the global coordinate system, $R_{1}=(G_{1},{\text{x}}^{\prime },\text{y},{\text{z}}^{\prime })$ is the body fixed coordinate system associated with body 1, $R_{2}=(A,{\text{x}}^{\prime \prime },\text{y},{\text{z}}^{\prime \prime })$ is the body fixed coordinate system associated with body 2. G_1_ is the centre of gravity of body 1 and G_2_ the centre of gravity of body 2. *m*_1_ and *m*_2_ are the masses of body 1 and 2, respectively. Point A is part of, in the sense that it moves with, body 2. The positions of G_1_, G_2_ and A are given in $R_{0}$ by the coordinate vectors **u**_G_1__, **u**_G_2__ and **u**_A_, respectively, with

2.1 $${\text{u}}_{G_{1}}={\text{OG}}_{\text{1}}=\left(\begin{array}{c} u_{G_{1}}\\ 0\\ w_{G_{1}} \end{array}\right),\;{\text{u}}_{G_{2}}={\text{OG}}_{\text{2}}=\left(\begin{array}{c} u_{G_{2}}\\ 0\\ w_{G_{2}} \end{array}\right)\;\mathrm{and}\;{\text{u}}_{A}=\text{OA}=\left(\begin{array}{c} u_{A}\\ 0\\ w_{A} \end{array}\right).$$

When the system is at rest, G_1_ and A coincide with O and **x**′ and **z**′ coincide with **x** and **z**, respectively. From the geometry of the system, at any time, vectors **G**_**1**_**A** and **z**′′ are always parallel. This geometric constrain can be expressed by

2.2 $${\text{G}}_{\text{1}}\text{A}\times {\text{z}}^{\prime \prime }=\text{0}.$$

The orientation of $R_{1}$ and of $R_{2}$ are given in $R_{0}$ by vectors *Ω*_1_ and *Ω*_2_, respectively, with

2.3 $$\Omega_{1}=\left(\begin{array}{c} 0\\ \theta \\ 0 \end{array}\right)\;\mathrm{and}\;\Omega_{2}=\left(\begin{array}{c} 0\\ \theta +\theta_{0}\\ 0 \end{array}\right),$$

where *θ* is the time varying angle between **x** and **x**′ and *θ*_0_ is the fixed angle between **z**′ and **z**′′.

### (b) Efforts applied to the system

The different stresses applied to the bodies are given using screw notations. An effort screw (or wrench) S consists of two vectorial quantities corresponding to a load: a force **F** and an associated moment **M**_*P*_ (expressed with respect to a point *P*) acting on the system:

$$S={\left\{\begin{array}{c} {\text{F}}_{S}\\ {\text{M}}_{S,P} \end{array}\right\}}_{P}.$$

For more details on this concept, see [22] (ch. 2 and 3) and [23].

Body 1 is subjected to the following stresses: weight, buoyancy, wave-induced loads and the linkage load applied by body 2 to body 1 through the damper. Some of the stresses induced by weight and buoyancy cancel each other out; the remaining hydrostatic loads are accounted for by a hydrostatic screw ($H_{1}$). Wave-induced loads are split into hydrodynamic radiation loads ($R_{1}$) and hydrodynamic wave exciting loads ($E_{1}$). The linkage load applied by body 2 to body 1 is noted $L_{2\to 1}$. Finally, the effort screw accounting for the external loads applied to body 1 is

2.4 $$T_{\mapsto 1}=E_{1}+R_{1}+H_{1}+L_{2\to 1.}$$

The linkage between body 1 and body 2 is of cylinder type which means that it has two degrees of freedom: translation along the **z**′′ direction and rotation around that same **z**′′ axis. Given that the motion of the system is restricted to the (**x**,**z**) plane the latter degree of freedom is irrelevant. The linkage is fitted with a linear damper along the **z**′′ direction.

2.5 $$L_{2\to 1}={\left\{\begin{array}{c} {\text{F}}_{L_{2\to 1}}\\ {\text{M}}_{L_{2\to 1}} \end{array}\right\}}_{O}={\left\{\begin{array}{c} ({\text{F}}_{L_{2\to 1}}\cdot {\text{x}}^{\prime \prime }){\text{x}}^{\prime \prime }+{\text{F}}_{\mathrm{dmp}}\\ {\text{M}}_{L_{2\to 1}} \end{array}\right\}}_{O},$$

where **F**_dmp_ is the force from the damper given by

2.6 $${\text{F}}_{\mathrm{dmp}}=-\alpha (({\dot{\text{u}}}_{G_{1}}-{\dot{\text{u}}}_{A})\cdot {\text{z}}^{\prime \prime }){\text{z}}^{\prime \prime },$$

with *α*≥0 being the damping coefficient.

Hydrodynamic and hydrostatic quantities are computed by WAMIT in G_1_ but need to be expressed with respect to O to solve the equations of motion. Let one consider $H_{1}$ in detail:

2.7 $$H_{1}={\left\{\begin{array}{c} {\text{F}}_{H_{1}}\\ {\text{M}}_{H_{1},G_{1}} \end{array}\right\}}_{G_{1}}={\left\{\begin{array}{c} {\text{F}}_{H_{1}}\\ {\text{M}}_{H_{1},G_{1}}+{\text{OG}}_{\text{1}}\times {\text{F}}_{H_{1}} \end{array}\right\}}_{O},$$

where **OG**_**1**_ is the vector from O to G_1_. Under the assumption of linearity, the magnitude of **OG**_**1**_ and of **F**_*H*_1__ are first-order small quantities. Their product is, therefore, a second-order small quantity and is thus negligible compared with **M**_*H*_1_,G_1__ and $H_{1}$ can be approximated as

2.8 $$H_{1}\approx {\left\{\begin{array}{c} {\text{F}}_{H_{1}}\\ {\text{M}}_{H_{1},G_{1}} \end{array}\right\}}_{O}.$$

Similarly

2.9 $$R_{1}\approx {\left\{\begin{array}{c} {\text{F}}_{R_{1}}\\ {\text{M}}_{R_{1},G_{1}} \end{array}\right\}}_{O}\;\mathrm{and}\;E_{1}\approx {\left\{\begin{array}{c} {\text{F}}_{E_{1}}\\ {\text{M}}_{E_{1},G_{1}} \end{array}\right\}}_{O}.$$

Body 2 is subjected to the following stresses: weight and the linkage stress applied by body 1 to body 2 through the damper ($L_{1\to 2}$). Since body 2 is a point mass without volume, it is not subjected to any hydrodynamic loads. However, it is assumed that its weight is compensated by a force equal in magnitude and opposite in direction which could be assimilated to buoyancy. As explained in the introduction, body 2 is an approximation of the set consisting of the piston and the body of water contained in the tube (figure 2*a*). The set has a density which is dominated by that of water and is therefore neutrally buoyant. This assumption is necessary to ensure that the two-body system is stable at rest. If the weight of body 2 was not compensated the system would sink. In consequence, the effort screw accounting for the external loads applied to body 2 is reduced to

2.10 $$T_{\mapsto 2}=L_{1\to 2}=-L_{2\to 1}.$$

### (c) Dynamics equations

The dynamic screw concept (or acceleration screw) D of a body is similar to that of wrench introduced in §2. It consists of two vectorial quantities: the linear acceleration and the angular acceleration (dynamic moment, noted ***δ***) given with respect to a point.

For body 1,

2.11 $$D_{1}={\left\{\begin{array}{c} m_{1}{\ddot{\text{u}}}_{G_{1}}\\ \delta_{1,G_{1}} \end{array}\right\}}_{G_{1}}={\left\{\begin{array}{c} m_{1}{\ddot{\text{u}}}_{G_{1}}\\ \delta_{1,G_{1}}+{\text{OG}}_{1}\times m_{1}{\ddot{\text{u}}}_{G_{1}} \end{array}\right\}}_{O}\approx {\left\{\begin{array}{c} m_{1}{\ddot{\text{u}}}_{G_{1}}\\ \delta_{1,G_{1}} \end{array}\right\}}_{O},$$

where the Newton's (or dot) notation is used for differentiation with respect to time. The approximation made in (2.11) is consistent with the assumption of linearity as explained for (2.8). Similarly for body 2,

2.12 $$D_{2}={\left\{\begin{array}{c} m_{2}{\ddot{\text{u}}}_{G_{2}}\\ \delta_{2,G_{2}} \end{array}\right\}}_{G_{2}}={\left\{\begin{array}{c} m_{2}{\ddot{\text{u}}}_{G_{2}}\\ \delta_{2,G_{2}}+{\text{OG}}_{2}\times m_{2}{\ddot{\text{u}}}_{G_{2}} \end{array}\right\}}_{O}.$$

Newton's second law for the two-body system is expressed by

2.13 $$D_{1}=T_{\mapsto 1}\;\mathrm{and}\;D_{2}=T_{\mapsto 2}.$$

Introducing the generalized displacement vector ***ξ***

2.14 $$\xi =\left(\begin{array}{c} \xi_{1}\\ \xi_{2}\\ \xi_{3}\\ \xi_{4}\\ \xi_{5} \end{array}\right)=\left(\begin{array}{c} u_{G_{1}}\\ w_{G_{1}}\\ \theta \\ u_{A}\\ w_{A} \end{array}\right),$$

(2.14) and (2.2) lead, in the frequency domain, to the following matrix equation whose detailed derivation is given in §A of the electronic supplementary material:

2.15 $$(-\omega^{2}(M+A^{H})+i\omega (B^{H}+B^{S})+(C^{H}+C^{S}))\xi ={\text{E}}^{H},$$

where *ω* is the radian frequency and with

2.16 $$\begin{array}{rl} M & =\left(\begin{array}{ccccc} m_{1} & 0 & m_{2}{w_{G_{2}}}_{r} & m_{2} & 0\\ 0 & m_{1} & -m_{2}{u_{G_{2}}}_{r} & 0 & m_{2}\\ 0 & 0 & I_{1,G_{1}}+m_{2}({u_{G_{2}}}_{r}^{2}+{w_{G_{2}}}_{r}^{2}) & m_{2}{w_{G_{2}}}_{r} & -m_{2}{u_{G_{2}}}_{r}\\ 0 & 0 & 0 & 0 & 0\\ 0 & 0 & -m_{2}{u_{G_{2}}}_{r}\cos\theta_{0}-m_{2}{w_{G_{2}}}_{r}\sin\theta_{0} & -m_{2}\sin\theta_{0} & m_{2}\cos\theta_{0} \end{array}\right), \end{array}$$

2.17 $$\begin{array}{rl} A^{H} & =\left(\begin{array}{ccccc} & & & 0 & 0\\ & A & & 0 & 0\\ & & & 0 & 0\\ 0 & 0 & 0 & 0 & 0\\ 0 & 0 & 0 & 0 & 0 \end{array}\right)\;\mathrm{and}\;B^{H}=\left(\begin{array}{ccccc} & & & 0 & 0\\ & B & & 0 & 0\\ & & & 0 & 0\\ 0 & 0 & 0 & 0 & 0\\ 0 & 0 & 0 & 0 & 0 \end{array}\right), \end{array}$$

2.18 $$\begin{array}{rl} B^{S} & =\left(\begin{array}{ccccc} 0 & 0 & 0 & 0 & 0\\ 0 & 0 & 0 & 0 & 0\\ 0 & 0 & 0 & 0 & 0\\ 0 & 0 & 0 & 0 & 0\\ \alpha \sin\theta_{0} & -\alpha \cos\theta_{0} & 0 & -\alpha \sin\theta_{0} & \alpha \cos\theta_{0} \end{array}\right), \end{array}$$

2.19 $$\begin{array}{rl} C^{H} & =\left(\begin{array}{ccccc} & & & 0 & 0\\ & C & & 0 & 0\\ & & & 0 & 0\\ 0 & 0 & 0 & 0 & 0\\ 0 & 0 & 0 & 0 & 0 \end{array}\right)\;\mathrm{and}\;C^{S}=\left(\begin{array}{ccccc} 0 & 0 & 0 & 0 & 0\\ 0 & 0 & 0 & 0 & 0\\ 0 & 0 & 0 & 0 & 0\\ -\cos\theta_{0} & -\sin\theta_{0} & 0 & \cos\theta_{0} & \sin\theta_{0}\\ 0 & 0 & 0 & 0 & 0 \end{array}\right) \end{array}$$

2.20 $$\begin{array}{rl} \text{and}\;{\text{E}}^{H} & =\left(\begin{array}{c} \text{E}\\ 0\\ 0 \end{array}\right), \end{array}$$

*u*_G_2___*r*_ and *w*_G_2___*r*_ are the coordinates in $R_{0}$ of G_2_ when the system is at rest. ***A***, ***B*** and ***C*** are, respectively, the added mass, hydrodynamic damping and hydrostatic 3×3 matrices computed by WAMIT. **E** is wave exciting force 3×1 vector also computed by WAMIT.

## 3. Verification of the numerical model

The above derivation is relatively complex and it is important to ensure that no mistake has been left unspotted before using the model for optimization purposes. To do so, the model needs to be verified, to make sure that its outcomes are consistent with the underlying theory, so as to show that the model is doing what it is supposed to do [24]. This is done by comparing model results with benchmark computations from WAMIT. The results are also checked to ensure that they make physical sense.

### (a) Comparison with benchmark data

**Figure 4.**
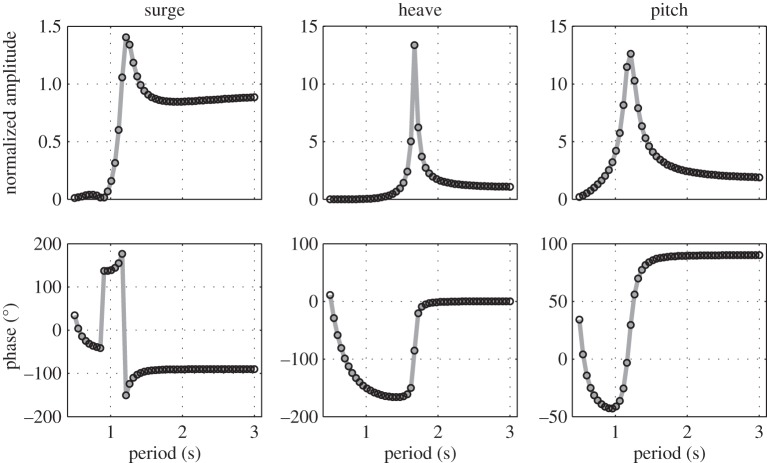
Comparisons between the response amplitude operators computed using only WAMIT with a modified mass matrix (grey line) and those calculated using equation (2.16) (black circle). Results are plotted as normalized amplitude (first row) and phase (second row) for the three degrees of freedom of interest.

In the model derived in §sec2, WAMIT is only used to compute the hydrodynamic and hydrostatic coefficients. However, WAMIT also includes a motion solver. As WAMIT is a widely used and thoroughly tested package, the results from its motion solver can be considered as numerical benchmark data. The WAMIT motion solver cannot deal with a point mass as one of the body of a multi-body system. However, if the damping coefficient of the damper is set to a very large value, there will hardly be any relative motion between the float and the point mass, and the two-body system (as shown in figure 2*b*) can be assimilated to a single body, which can be modelled by WAMIT. The results from the model derived in this paper are therefore compared with those of a WAMIT single-body simulation but for which the mass properties of that single-body account for both the float and the point mass. The comparison is shown in figure 4. For this simulation, the float is a cylinder 0.5 m in diameter with a 0.5 m draft, its mass is *m*_1_=98.17 kg and its centre of gravity is located on the cylinder axis, 0.4 m below the mean water level. The point mass is located 0.1 m below the centre of gravity of the float and its mass is a-tenth of that of the float: *m*_2_=*m*_1_/10=9.82 kg. The damping coefficient *α*=10^9^ Nsm^−1^ and the damper angle *θ*_0_=0°. The response amplitudes shown in figure 4 are normalized by wave amplitude for surge and heave, and by wave slope (defined as arctan⁡((2×wave amplitude)/(wave length/2))) for pitch.

The match between the two computations is excellent for all degrees of freedom, in terms of both amplitude and phase. The discrepancies do not exceed 7.67×10^−4^ in amplitude. Similar results are observed for any value of *θ*_0_. This contributes to build confidence in the model derivation.

### (b) Physical interpretation of simulations

Another way of providing reassurance in the model derivation is to gradually increase the values of the damping coefficient and of the mass of the point mass and to check that the evolution of the bodies response makes physical sense.

**Figure 5.**
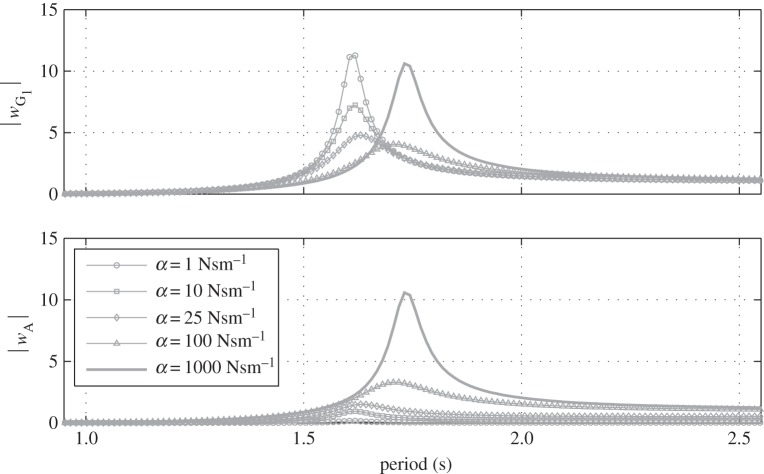
Normalized amplitude of the heave displacement of the float (*w*_G_1__) and of the point mass (*w*_A_) for various values of the damping coefficient *α*.

Figure 5 shows the amplitude of the heave response of the float (*w*_G_1__) and of the point mass (*w*_A_) for increasing values of the damping coefficient *α*, ranging from 1 to 1000 Nsm^−1^. In comparison, the maximum radiation damping values of the float in surge and in heave are 333 Nsm^−1^ and 15 Nsm^−1^, respectively.

It can be seen that for low damping values the heave motion of the point mass is small and that it increases with increasing damping values. This makes physical sense as with low values of *α*, both bodies are hardly coupled and given that the point mass is not subjected to any hydrodynamic or hydrostatic load, there is very little force to excite its motion. Conversely, when the damping is at its highest, there can hardly be any relative motion between the two bodies, hence the similar heave response of the float and of the point mass. It can also be seen that as damping increases up to 100 Nsm^−1^, the amplitude of motion of the float at resonance is reduced whereas for the much higher damping value of 1000 Nsm^−1^, the float motion is large again. This can be explained by the fact that for damping values up to 100 Nsm^−1^, some of the energy in the system is dissipated by the damper which has the effect of reducing the float motion amplitude. For the highest damping value, however, there is hardly any relative motion between both bodies and very little energy is dissipated by the damper hence the large motion of the float. It can also be observed that the resonance period of the heave response increases with *α*. This can be physically explained by the fact that for low damping there is little coupling between the two bodies and the heave resonance period *T*_*h*_res__ of the float is mainly driven by the square root of the ratio of its mass *m*_1_ plus heave added mass *a*_*h*_ over the heave hydrostatic stiffness *c*_*h*_: $T_{h_{\mathrm{res}}}\propto \sqrt{(m_{1}+a_{h})/c_{h}}$. However, for the highest damping value, the point mass can be considered integral to the float and is increasing its overall inertia. Given that the heave hydrostatic stiffness remains unchanged because it only depends on the geometry of the float, the heave resonance period increases: $T_{h_{\mathrm{res}}}\propto \sqrt{(m_{1}+a_{h}+m_{2})/c_{h}}$. Variations in *α* affect the system response in heave but not in surge or in pitch and that is why these latter degrees of freedom are not plotted in figure 5. Given that the damper is vertical (*θ*_0_=0°), the system is symmetrical about a vertical plane which is perpendicular to the direction of propagation of the waves. In these conditions, the heave mode is decoupled from surge and pitch (see [25], p. 308 for more detail).

**Figure 6.**
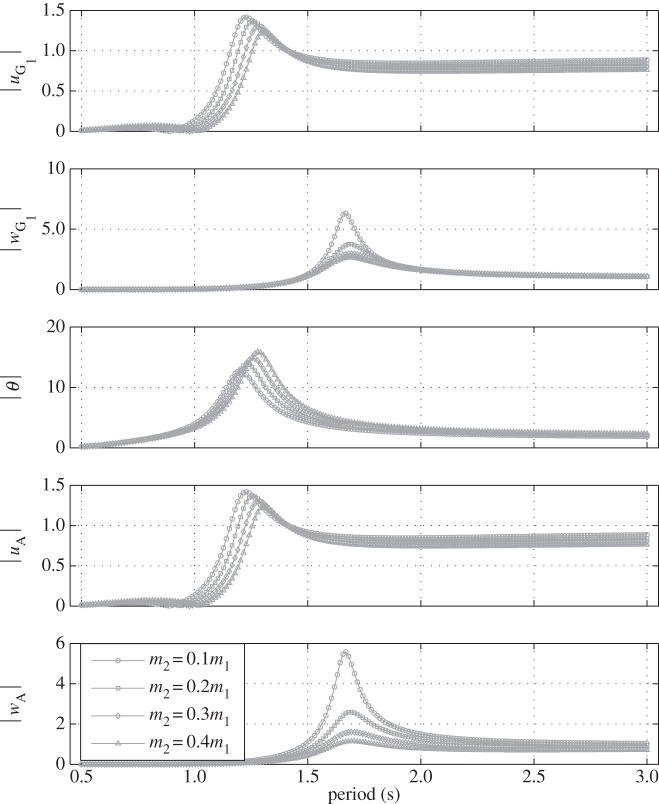
Normalized amplitude of the displacements of the float (*u*_G_1__, *w*_G_1__ and *θ*) and of the point mass (*u*_A_ and *w*_A_) with the damper set vertical (*θ*_0_=0°) and *α*=70 Nsm^−1^ for various mass values of the point mass.

In figure 6, the float geometry, its mass properties and the position of the point mass are the same as before. The damper is set vertical (*θ*_0_=0°), the damping value is fixed (*α*=70 Nsm^−1^) and the impact of increasing the mass *m*_2_ of the point mass is shown. In heave, it can be seen that the amplitude of the response of the point mass (*w*_A_) decreases with increasing values of *m*_2_. This makes physical sense given that the inertia of the point mass increases with *m*_2_ whereas the exciting force it experiences from the float via the damper remains largely unchanged. It can also be seen that the resonance period of the float response in heave (*w*_G_1__) increases slightly with *m*_2_. This is to be expected given that, in that mode of motion there is coupling between the two bodies (via the damper) and that therefore an increase in *m*_2_ tends to increase the heave inertia of the overall system whereas the hydrostatic stiffness remains the same. In surge and pitch (which are coupled), it can be observed that the resonance period also increases with *m*_2_ but more significantly than for heave. An increase in *m*_2_ brings about an increase in the pitch moment of inertia whereas the hydrostatic restoring torque stays constant. As for heave, this has the effect of increasing the resonance period. Moreover, given that the damper is vertical, the float and the point mass can be considered to be rigidly connected in surge and pitch and so the increase in *m*_2_ impacts directly on float surge and pitch inertia and not in a ‘damped’ manner as it is the case in heave.

**Figure 7.**
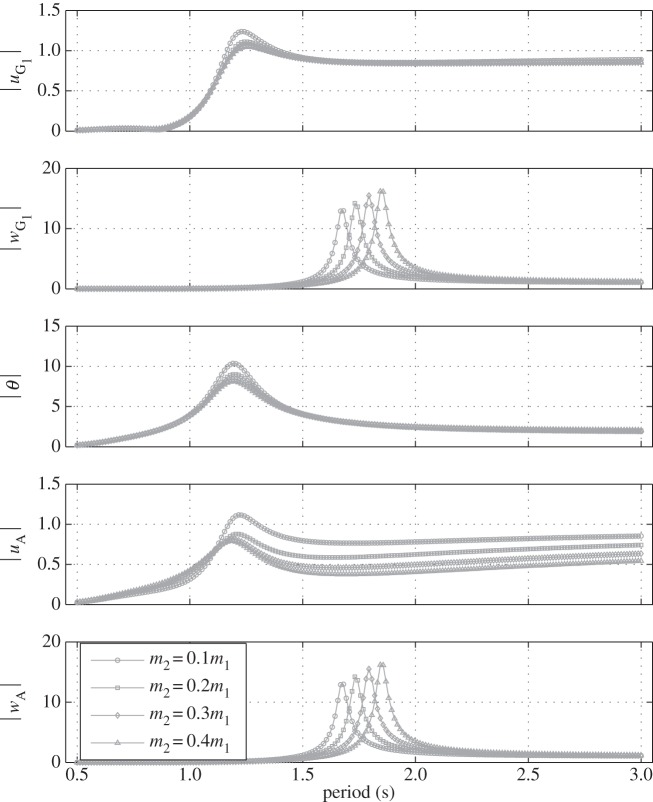
Normalized amplitude of the displacements of the float (*u*_G_1__, *w*_G_1__ and *θ*) and of the point mass (*u*_A_ and *w*_A_) with the damper set horizontal (*θ*_0_=90°) and *α*=70 Nsm^−1^ for various mass values of the point mass.

Figure 7 shows the response of the system in the same conditions as in figure 6 except that the damper is set horizontal (*θ*_0_=90°). It can be seen that in this configuration, the heave motion of the float (*w*_G_1__) is the same as the heave motion of the point mass (*w*_A_). The impact of increased *m*_2_ on the heave resonance period is also more pronounced than in figure 6. This is to be expected given that with the damper horizontal, the float and the point mass can be considered to be rigidly connected in heave. The amplitude of the pitch response is lower than in figure 6, which can be explained by the fact that with the damper horizontal, the pitch motion is now damped. This builds confidence in the way the damper and the angle at which its sits are modelled.

In figures 6 and 7, it can be seen that for long wave periods, the heave response amplitude of the float converges towards 1. This is consistent with the fact that in those circumstances, the heave wave loads are dominated by hydrostatic terms and the float therefore follows the wave surface (see [26], p. 134 for more details).

## 4. Parametric study and optimization

Figures 4–7 and their interpretations provide a strong verification of the model and build the necessary confidence to be able to use the model reliably for parametric study and optimization purposes. The WEC configuration considered in this context consists of a point mass and of the same float as the one described in §sec3a. The mass properties of the float are fixed and are also described in §sec3a. The point mass is neutrally buoyant (as explained in §sec2b) and it is located along the vertical axis of the cylindrical float when the system is at rest.

The geometry and mass properties of the float itself have deliberately been excluded from the optimization process. Such shape optimization studies have already been reported in the literature [27–30]. The present investigation focuses instead on PTO parameters with an emphasis on sloped motion.

No scale factor or any particular sea state are specified for the optimization. Doing so would have inevitably made the device size a key parameter for performance. This would have in turn shifted the focus of the study away from investigating purely the sloped PTO.

### (a) Scopes and metrics

The parameters investigated and their range are given by table 1. It should be noted that while *m*_2_ was allowed to vary between [0.2*m*_1_ 1.2*m*_1_], the optimization will only be conducted with values of *m*_2_≤*m*_1_. Extending the range of *m*_2_ helps ensuring the validity of the models for values of *m*_2_ close to *m*_1_. The upper *m*_2_ value retained for the optimization (*m*_2_=*m*_1_) would correspond to a tube whose internal volume is similar to that of the float. Although detailed engineering considerations are beyond the scope of this study, it is believed that this can be achieved, by spreading the reference body of water across several PTO tubes, for example. The *w*_G_2___*r*_ range was chosen so that the point mass is always below the centre of gravity of the float and therefore does not reduce its stability. It can be noticed that the range of *w*_G_2___*r*_ starts at −0.05 *m*, which is within the float and not below it. From an engineering point of view, this would correspond to having the PTO tube integrated to the float. The *w*_G_2___*r*_ lower limit was set to −0.5×cylinder draft as too large a distance between the float and the tube could be impractical from an engineering perspective.

**Table 1.** Description and range of the parameters investigated for parametric study and optimization. The last column expresses the range of some of the parameters as a function of the float characteristics.

parameter	symbol	range	range (relative value)
mass of the point mass	*m*_2_	[19.6–117.8] kg	[0.2*m*_1_–1.2*m*_1_]
vertical position of the point mass with respect to the float	*w*_G_2___*r*_	[−0.05 to −0.25] m	[−0.1 to −0.5]×cylinder draft
angle of the PTO	*θ*_0_	[0–90]°	
PTO damping	*α*	[100–1000] Nsm^−1^	

Two metrics are considered to assess the performance of the WEC configurations explored. They are derived from the capture width ratio (*CW*) which corresponds to the ratio of the energy harvested by the WEC over the incoming wave energy available in a wave front whose width is equal to that of the device. More information on that concept can be found in [31].

The power captured by the WEC is calculated from the relative translational motion between the float and the point mass along the damper direction. The velocity associated with that motion is multiplied by the PTO damping force to obtain the captured power.

The two metrics used to assess WEC performance are the following.

*Capture width area* (CW_area_) corresponds to the capture width ratio integrated over the wave period spectrum. Physically, it gives an indication of the energy capture performance over the whole spectral range.
*Mean capture width period* (*T*_*m*_CW) is the mean of the wave period weighted by the capture width ratio:
  4.1 $$T_{m}\mathrm{CW}=\frac{1}{{\mathrm{CW}}_{\mathrm{area}}}\int_{\mathrm{spectrum}}\mathrm{CW}(T)T\;dT,$$
  where *T* is the wave period and CW(*T*), the capture width ratio value at that wave period. Physically, this metric provides an indication of the ability of a configuration to perform well in the more energetic longer waves. As the geometry of the float is fixed, good performance in long waves is advantageous because it potentially increases power production while keeping the float size, and therefore to some extends its cost, constant.

### (b) Method

#### (i) Generation of data

Four thousand WEC configurations are generated by randomly selecting 4000 combinations of the four parameters within the range specified in table 1. The performance and body motions of each configuration are then computed over a 0.5 to 4 s wave period range using the equations derived in §sec2c.

The amplitude of motions predicted by linear BEM can be non-physically large. This is mainly due to the fact that potential flow theory, on which BEM is based, does not account for viscous effects which damp body motion. Unrealistically large body motions can lead to erroneous predictions of WEC power absorption which would in turn distort the PTO optimization. To circumvent this issue, configurations exhibiting normalized motion amplitudes (as defined in §sec3a) higher than 10 are discarded. This way, from an initial set of 4000 randomly generated WEC configurations, 600 are typically retained.

#### (ii) Scores

The optimization process with respect to each of the metrics defined in §sec4a taken individually could lead to conflicting requirements. A way around this issue is to combine the different metrics in a single objective function leading to a single score. This is done as follows. For a given WEC configuration, each metric is normalized by its maximum value achieved over the full set of retained configurations. The score of each configuration is then defined as the weighted sum of the normalized metrics, keeping the sum of the weight equal to 1. This ensures that scores are kept within the [0 1] range. This approach makes it possible to control and investigate the influence of each metric on the overall score. Three weight combinations are defined as part of the optimization process:

4.2 $$\begin{array}{rl} {\mathrm{score}}_{20} & =0.2\frac{T_{m}\mathrm{CW}}{\max(T_{m}\mathrm{CW})}+0.8\frac{{\mathrm{CW}}_{\mathrm{area}}}{\max({\mathrm{CW}}_{\mathrm{area}})} \end{array}$$

4.3 $$\begin{array}{rl} {\mathrm{score}}_{40} & =0.4\frac{T_{m}\mathrm{CW}}{\max(T_{m}\mathrm{CW})}+0.6\frac{{\mathrm{CW}}_{\mathrm{area}}}{\max({\mathrm{CW}}_{\mathrm{area}})} \end{array}$$

4.4 $$\begin{array}{rl} \text{and}\;\;{\mathrm{score}}_{50} & =0.5\frac{T_{m}\mathrm{CW}}{\max(T_{m}\mathrm{CW})}+0.5\frac{{\mathrm{CW}}_{\mathrm{area}}}{\max({\mathrm{CW}}_{\mathrm{area}})} \end{array}$$

#### (iii) Optimization

The process of optimization consists first in fitting an analytical model to the observed score distribution. The parameters of the model (defined in table 1) are the variables to optimize. The scores and associated variables (*m*_2_,*w*_G_2___*r*_,*θ*_0_ and *α*) are imported into the statistical analysis package R [32].

### (c) Results

#### (i) Experimental plan

**Figure 8.**
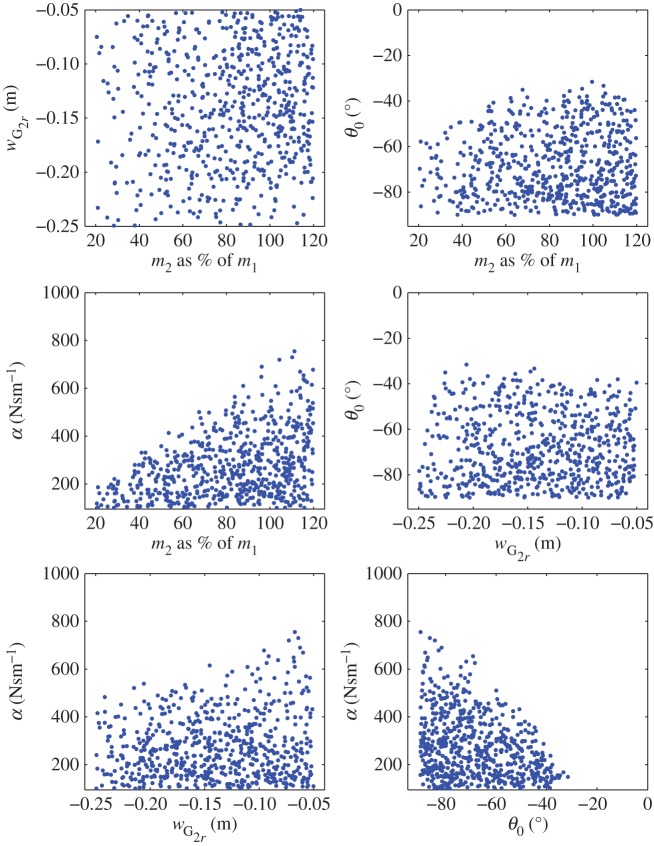
Resulting experimental plan obtained after excluding configurations for which motion amplitudes were dimmed unrealistic.

Figure 8 shows the resulting experimental plan obtained after discarding WEC configurations with unrealistic motion responses. Some clear boundaries can be observed; in particular, the one limiting the range in which *α* can evolve as a function of the three other variables to optimize. Any further results of the optimization would have to be located within the bounds of the experimental plan.

It can be noted that the nearest angle to vertical included in the experimental plan is about *θ*_0_=−30°. Indeed, for PTO angles closer to vertical, the normalized pitch motion exceeds 10. Although these large pitch responses are partly due to the numerical model neglecting viscous and radiation damping on the PTO structure, it also reflects a real physical pitch instability exhibited by some free-floating WECs with similar geometry and a vertical PTO [33]. In this context, a sloped PTO direction brings beneficial damping to the pitch motion.

#### (ii) R model

**Figure 9.**
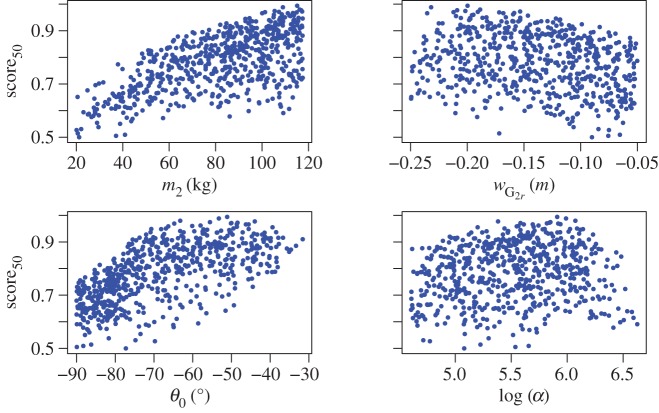
score_50_ with respect to the four parameters to optimize. log⁡(α) is used instead of *α* as it was found to better spread the data and to lead to a better model fit.

Figure 9 shows score_50_ plotted as a function of the four variables. log⁡(α) is used instead of *α* as it provides a better spread of the points with respect to that variable. It can be seen from these plots that linear trends of score_50_ with respect to *m*_2_ and *w*_G_2___*r*_ can be expected, whereas at least square laws should be considered to model the evolution of score_50_ with respect to *θ*_0_ and log⁡(α).

The model is fitted to the distribution in R using the following call:

   logModel <- lm(score50 ∼ {I}(theta0^2) + I((log(alpha))^2) +  (theta0 + m2 + alpha + wG2r)^2).

This model considers a square term for *θ*_0_ and log⁡(α), as well as linear terms for the four variables and their possible interactions.

The model is then simplified to remove the non-significant terms. The final model fitted to score_50_ is presented in equation (4.5).

4.5 $$\begin{array}{rl} {\mathrm{score}}_{50}(m_{2},\theta_{0},\alpha ,{w_{G_{2}}}_{r}) & =-1.614-0.1641e^{-3}\theta_{0}^{2}-0.06479\log{\left(\alpha \right)}^{2}-0.01809\theta_{0}\\ & \;+0.2134e^{-2}m_{2}+0.6276\log(\alpha )+0.2093e^{-4}\theta_{0}m_{2}-0.3209e^{-3}\theta_{0}\log(\alpha )\\ & \;-0.5970e^{-2}\theta_{0}{w_{G_{2}}}_{r}+0.4651e^{-3}m_{2}\log(\alpha )+0.2867e^{-2}m_{2}{w_{G_{2}}}_{r}\\ & \;-0.2419\log(\alpha ){w_{G_{2}}}_{r}. \end{array}$$

The summary of the models and the check plots are presented in §B of the electronic supplementary material. It can be observed that the model is not perfect, as the residuals appear to depart from normality. However, the fitted model is not to be used for extrapolation but solely for interpolation, and to reveal trends about the influence of each parameter. The *r*^2^-value associated to the model is 0.9674 and its residual standard error is 0.01909, which is thought to be acceptable given the observed dispersion.

Similar models were fitted to score_20_ and score_40_. These are summarized in §B of the electronic supplementary material.

#### (iii) Optimization results

Optimizing the WEC now corresponds to finding the combination of parameters maximizing the model scores. For score_50_, it can be observed that the model derivative with respect to *m*_2_ is positive over the entire experimental plan, whereas the model derivative with respect to *w*_G_2___*r*_ is always negative. This means that their optimal values are, respectively, the upper and lower bounds of their respective range (*m*_2_=*m*_1_ and *w*_G_2___*r*_=−0.25 m).

The optimal values for *θ*_0_ and *α* are found by solving the following system of equations:

4.6 $$\begin{array}{rl} \frac{\partial \;{\mathrm{score}}_{50}(m_{1},\theta_{0},\alpha ,-0.25)}{\partial \theta_{0}} & =0\\ \frac{\partial \;{\mathrm{score}}_{50}(m_{1},\theta_{0},\alpha ,-0.25)}{\partial \alpha } & =0 \end{array}\}.$$

Solving (4.6) leads to *θ*_0_=−49.97° and *α*=325.76 Nsm^−1^. These values are within the bounds shown in figure 8. For this set of values, the score obtained from the analytical model is larger than 1: score_50_(*m*_2_=*m*_1_,*θ*_0_=−49.97,*α*=325.76,*w*_G_2___*r*_=−0.25)=1.011230516. By inputting these values of parameters to the hydrodynamic model derived in §sec2c, score_50_=0.9670 is obtained. By comparison, the highest score_50_ in the randomly generated data set with *m*_2_≤*m*_1_ is score_50_=0.9473. This means that the optimization method used has provided a combination of parameters with a better score than the one associated with any of the randomly generated configurations.

**Figure 10.**
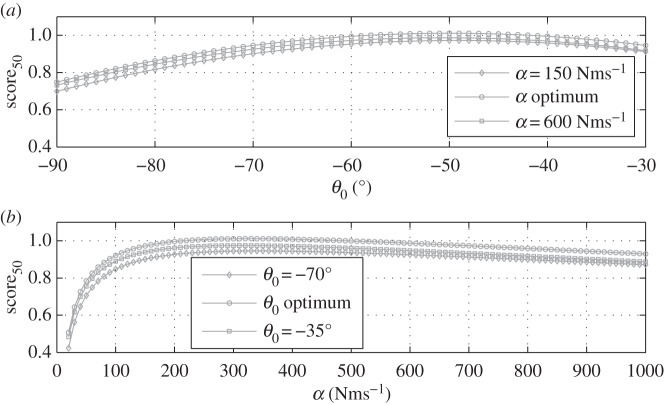
Variation of the analytical model fitted to score_50_ with respect to *θ*_0_ for three levels of PTO damping (*a*) and with respect to *α* for three values of *θ*_0_ (*b*). For both graphs *m*_2_ and *w*_G_2___*r*_ are set to optimum.

It is interesting to look at the sensitivity of score_50_ to *θ*_0_ and *α*. Figure 10 shows the variation of the analytical model as a function of *θ*_0_ for three levels of PTO damping and as a function of *α* for three values of *θ*_0_. It can be observed that the model shows a strong sensitivity to *θ*_0_. On the top plot, score_50_ increases rapidly as the PTO direction departs from horizontal towards a sloped angle, which suggests strong benefit in inclining the PTO direction. Low values of *α* are associated with very low scores. However, as soon as values over 100 are reached, the sensitivity to *α* is less pronounced. This suggests that it should be possible to vary significantly the PTO damping if required (e.g. for stability reasons or to limit the damper motion) without impacting too much on performance.

Finally, the influence of the weighting factors used for the definitions of the *scores* on the optimization results is investigated. The optimal values for *m*_2_ and *w*_G_2___*r*_ are identical in the three *scores* defined in §sec4b(ii). Table 2 gives a summary of the optimum configurations corresponding to the three different scores.

**Table 2.** Summary of the optimal configurations found for the three different scores defined in §sec4b(ii). From the left to the right, relative importance of CW_area_ is increasing and relative importance of *T*_*m*_CW is decreasing.

	optimum score_50_	optimum score_40_	optimum score_20_
*m*_2_	*m*_1_	*m*_1_	*m*_1_
*w*_G_2___*r*_	−0.25	−0.25	−0.25
*θ*_0_	−49.96	−49.87	−49.80
*α*	325.76	326.95	293.11
CW_area_	1.573	1.573	1.569
*T*_*m*_CW	1.808	1.808	1.801
score_50_	0.9668	0.9669	0.9639
score_40_	0.962	0.9621	0.9592
score_20_	0.9523	0.9524	0.9498

It can be observed that the optimal *θ*_0_ is very similar for the three different scores. This is not a parameter easy to adjust once a WEC has been built, and therefore having a well-defined and stable optimum is desirable. On the other hand, these results suggests that the optimum values for *α* might be more dependent on the selected weighting factors. The PTO damping is commonly a variable that can be adjusted on the device, and therefore its variability is less problematic. Additionally, as can be seen in figure 10, the sensitivity of the *scores* to *α* is low in the [250−400] Nsm^−1^ range, and the variability seen in table 2 might therefore not be in fact very significant.

Table 2 shows the capture width area, the mean capture width period and the different scores for each optimum. From these data, it can be seen that some configurations optimized for a given score perform less well for that score (by a tiny amount) than configurations optimized for a different score. For example, the optimum solution for score_20_ would be expected to have a higher score_20_ than the two other solutions but that is not the case. As the three solutions are very close, these discrepancies are thought to be due to noise in the optimization process. These discrepancies would be less likely to occur if the two optimization metrics did not lead to similar results as is the case here.

**Figure 11.**
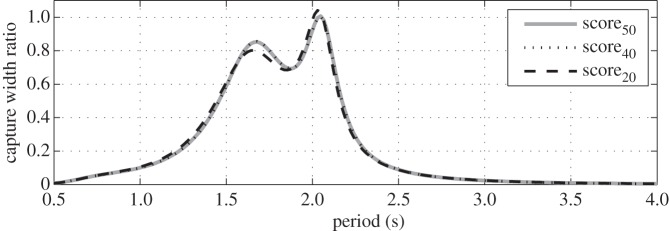
Capture width ratios plotted against wave period for the three *scores* investigated.

Figure 11 shows the capture width ratio as a function of wave period for the optimum configurations corresponding to the three *scores* investigated. As mentioned earlier the parameter values for the three *scores* are quite similar and hence so are the curves. The curves exhibit a ‘double peak’. From a hydrodynamics point of view, the first peak corresponds to the system resonance in pitch and the second one to the resonance in heave. A clear advantage of the sloped PTO configuration is to be able to harness energy from both pitch/surge and heave motions. Moreover, the capture width ratio curve only dips slightly between the two peaks, providing the device with a wide bandwidth of high capture with ratio (above 0.7). This feature is advantageous in that it allows the system to perform efficiently over a broad range of sea states without the need for complex control. In comparison, a purely heaving system has a narrower bandwidth.

## 5. Conclusion

The concept of inclining the PTO axis of a free-floating WEC reacting against water inertia has been investigated using numerical modelling. The shape of the WEC has deliberately been kept to that of a simple vertical axis circular cylinder and the study has focused on PTO parameters. The derivation of the numerical modelling has been presented in detail. The thorough validation of that model has been carried out by ensuring that the results made physical sense and by comparing these with benchmark numerical data. This successful validation has built the necessary confidence into the model so that it could reliably be used for the parametric study and the optimization which has investigated the impact of the mass of the point mass, of its vertical position, of the angle of the PTO damper and of the damping value on the WEC performance. A multi-linear regression method has been used to find the set of parameters which provides the best performance in terms of linear combinations of capture width area and mean capture width period. Four thousand WEC configurations have been generated in that process. The optimum values for *m*_2_ is the maximum of the range considered, i.e. *m*_2_=*m*_1_. For *w*_G_2___*r*_ it is the lowest value of the range: *w*_G_2___*r*_=−0.25 m. For the PTO angle and damping, the optimum values vary slightly with the score chosen. It is respectively between 293 and 327 Nms^−1^ and around 50°.

Given the simplicity of the WEC shape and the fact that the numerical modelling technique used is frequency domain BEM, computations for each configuration was quick. This makes it possible to compute the performance of a large number of configurations. In that context, it would have been possible to identify a best performing configuration by carrying out a systematic exploration of the parametric domain with a fine parametric resolution. It should, however, be noted that the fitting of the analytical model and the subsequent optimization did yield some performance improvement over the best of the 4000 initial cases, which is already a large number of configurations. But perhaps more importantly, the optimization approach used here has provided an understanding of the impact of each parameter on the device performance and behaviour and this could not have been achieved with a systematic ‘brute-force’ optimization.

The parametric study shows, in a comprehensive manner, the clear advantage of an inclined PTO direction versus a vertical one. This is physically due to the fact that inclined configurations exploit resonance in both heave and pitch, thus widening the period range over which high capture width ratios are observed. These fundamental results pave the way for exploring further WEC concepts featuring sloped PTOs.

## Supplementary Material

Electronic Supplementary Material, On the concept of sloped motion for free-floating wave energy converters

## Data accessibility

This article is solely based on numerical modelling and has therefore no data associated with it.
