# Supplementary material for: On the concept of sloped motion for free-floating wave energy converters
Source: Proc Math Phys Eng Sci. 2015 Oct 8;471(2182):20150238. doi: 10.1098/rspa.2015.0238 (PMC4633885; doi:10.1098/rspa.2015.0238)
Supplement: Electronic Supplementary Material, On the concept of sloped motion for free-floating wave energy converters [file rspa20150238supp1.pdf]

Electronic Supplementary Material

On the concept of sloped motion for free-floating wave energy converters

rspa.royalsocietypublishing.org Proc R Soc A

A. Equations of motion

Effort screws (or dynamic screws) can be expressed in vector form. In the general case of a six degrees of freedom motion, a screw is a six component column vector. The first three correspond to the force and the remaining three correspond to the moment. Given that only surge, heave and pitch are considered (see section 2(a) of the main body of the article), a screw can be expressed by a vector with only three components:

$$\mathcal{S} = \begin{pmatrix} F_x \\ F_z \\ M_y \end{pmatrix} \tag{A 1}$$

(a) Dynamic screws

$\mathcal{D}_1$ introduced in (2.11) can be expressed by the vector:

$$\mathcal{D}_1 = \begin{pmatrix} m_1 \ddot{u}_{G_1} \\ m_1 \ddot{w}_{G_1} \\ I_{1,G_1} \ddot{\theta} \end{pmatrix} \tag{A 2}$$

where I_{1,G_1} is the moment of inertia of body 1 along the y-axis expressed in G_1.

The derivation of $\mathcal{D}_2$ introduced in (2.12) requires the derivation of $\ddot{\mathbf{u}}_{G_2}$ which can be obtained from $\dot{\mathbf{u}}_{G_2}$.

$$\begin{aligned} \dot{\mathbf{u}}_{G_2} &= \dot{\mathbf{u}}_A + \mathbf{G_2A} \times \dot{\Omega}_2 \\ &= \dot{\mathbf{u}}_A + (\mathbf{G_2O} + \mathbf{OA}) \times \dot{\Omega}_2 \\ &\approx \dot{\mathbf{u}}_A - \mathbf{OG_2} \times \dot{\Omega}_2 \end{aligned} \tag{A 3}$$

At first order,

$$\dot{\mathbf{u}}_{G_2} = \begin{pmatrix} \dot{u}_A + w_{G_2}\dot{\theta} \\ 0 \\ \dot{w}_A - u_{G_2}\dot{\theta} \end{pmatrix} \tag{A 4}$$

The position of point G_2 varies with time but the coordinates of G_2 can be expressed as follows:

$$\begin{pmatrix} u_{G_2} \\ 0 \\ w_{G_2} \end{pmatrix} = \begin{pmatrix} u_{G_2 r} + \Delta u_{G_2} \\ 0 \\ w_{G_2 r} + \Delta w_{G_2} \end{pmatrix} \tag{A 5}$$

$u_{G_2 r}$ and $w_{G_2 r}$ are the coordinates of G_2 when the system is at rest. $u_{G_2 r}$ and $w_{G_2 r}$ are therefore constant. Δu_{G_2} and Δw_{G_2} express the displacement of G_2 from its rest position. Under the assumption that motions are small with respect to body overall dimensions and to wavelength, Δu_{G_2} and Δw_{G_2} are first order time varying small quantities. The derivation of $\dot{\mathbf{u}}_{G_2}$ at first order therefore yields:

$$\ddot{\mathbf{u}}_{G_2} = \begin{pmatrix} \ddot{u}_A + w_{G_2 r}\ddot{\theta} \\ 0 \\ \ddot{w}_A - u_{G_2 r}\ddot{\theta} \end{pmatrix} \tag{A 6}$$

and

$$\mathcal{D}_2 = \begin{pmatrix} m_2\left(\ddot{u}_A + w_{G_2 r}\ddot{\theta}\right) \\ m_2\left(\ddot{w}_A - u_{G_2 r}\ddot{\theta}\right) \\ I_{2,G_2}\ddot{\theta} + m_2\left((u_{G_2 r}^2 + w_{G_2 r}^2)\ddot{\theta} - u_{G_2 r}\ddot{w}_A + w_{G_2 r}\ddot{u}_A\right) \end{pmatrix} \tag{A 7}$$

Body 2 is a point mass and therefore $I_{2,G_2} = 0$, hence:

$$\mathcal{D}_2 = \begin{pmatrix} m_2\left(\ddot{u}_A + w_{G_2 r}\ddot{\theta}\right) \\ m_2\left(\ddot{w}_A - u_{G_2 r}\ddot{\theta}\right) \\ m_2\left((u_{G_2 r}^2 + w_{G_2 r}^2)\ddot{\theta} - u_{G_2 r}\ddot{w}_A + w_{G_2 r}\ddot{u}_A\right) \end{pmatrix} \tag{A 8}$$

(b) Linkage force

The damper force derived in (2.6) can be expressed at first order by:

$$\mathbf{F}_{dmp} = -\alpha\left((\dot{w}_{G_1} - \dot{w}_A)\cos\theta_0 - (\dot{u}_{G_1} - \dot{u}_A)\sin\theta_0\right)\mathbf{z}'' \tag{A 9}$$

Expressed along $\mathbf{x}$ and $\mathbf{z}$ at first order, (A 9) becomes:

$$\begin{aligned} \mathbf{F}_{dmp} = &-\alpha\left((\dot{u}_{G_1} - \dot{u}_A)\sin^2\theta_0 - (\dot{w}_{G_1} - \dot{w}_A)\cos\theta_0\sin\theta_0\right)\mathbf{x} \\ &- \alpha\left(-(\dot{u}_{G_1} - \dot{u}_A)\sin\theta_0\cos\theta_0 + (\dot{w}_{G_1} - \dot{w}_A)\cos^2\theta_0\right)\mathbf{z} \end{aligned} \tag{A 10}$$

$\left(\mathbf{F}_{L_{2\to1}} \cdot \mathbf{x}''\right)\mathbf{x}''$ introduced in (2.5) expressed along $\mathbf{x}$ and $\mathbf{z}$ yields at first order:

$$\left(\mathbf{F}_{L_{2\to1}} \cdot \mathbf{x}''\right)\mathbf{x}'' = \left(\mathbf{F}_{L_{2\to1}} \cdot \mathbf{x}''\right)\cos\theta_0\mathbf{x} + \left(\mathbf{F}_{L_{2\to1}} \cdot \mathbf{x}''\right)\sin\theta_0\mathbf{z} \tag{A 11}$$

Eventually, $\mathcal{L}_{2\to1}$ introduced in (2.5) can be expressed by the vector:

$$\mathcal{L}_{2\to1} = \begin{pmatrix} \left(\mathbf{F}_{L_{2\to1}} \cdot \mathbf{x}''\right)\cos\theta_0 - \alpha\left((\dot{u}_{G_1} - \dot{u}_A)\sin^2\theta_0 - (\dot{w}_{G_1} - \dot{w}_A)\cos\theta_0\sin\theta_0\right) \\ \left(\mathbf{F}_{L_{2\to1}} \cdot \mathbf{x}''\right)\sin\theta_0 - \alpha\left(-(\dot{u}_{G_1} - \dot{u}_A)\sin\theta_0\cos\theta_0 + (\dot{w}_{G_1} - \dot{w}_A)\cos^2\theta_0\right) \\ M_{L_{2\to1}} \end{pmatrix} \tag{A 12}$$

where $M_{L_{2\to1}}$ is the such that $\mathbf{M}_{L_{2\to1}} = M_{L_{2\to1}}\mathbf{y}$

(c) Geometric constrain

Equation (2.2) which corresponds the geometric constrain of the system can be expressed as follow:

$$\begin{aligned} (-\mathbf{OG_1} + \mathbf{OA}) \times \mathbf{z}'' &= \begin{pmatrix} 0 \\ -(w_A - w_{G_1})\sin(\theta + \theta_0) - (u_A - u_{G_1})\cos(\theta + \theta_0) \\ 0 \end{pmatrix} \\ &= \mathbf{0} \end{aligned} \tag{A 13}$$

After linearisation this yields:

$$(w_{G_1} - w_A)\sin\theta_0 = (u_A - u_{G_1})\cos\theta_0 \tag{A 14}$$

(d) Equations of the system in the frequency domain

Newton's second law applied to the system, as expressed by equation (2.13) yields, in the frequency domain, the following six equations:

$$\begin{aligned}-m_1\omega^2 u_{\mathrm{G}_1} = &(\mathbf{F}_{E_1} + \mathbf{F}_{R_1} + \mathbf{F}_{H_1}) \cdot \mathbf{x} + \left(\mathbf{F}_{L_{2\to1}} \cdot \mathbf{x}''\right)\cos\theta_0 \\ &- i\omega\alpha\left((u_{\mathrm{G}_1} - u_{\mathrm{A}})\sin^2\theta_0 - (w_{\mathrm{G}_1} - w_{\mathrm{A}})\cos\theta_0\sin\theta_0\right)\end{aligned} \tag{A 15}$$

$$\begin{aligned}-m_1\omega^2 w_{\mathrm{G}_1} = &(\mathbf{F}_{E_1} + \mathbf{F}_{R_1} + \mathbf{F}_{H_1}) \cdot \mathbf{z} + \left(\mathbf{F}_{L_{2\to1}} \cdot \mathbf{x}''\right)\sin\theta_0 \\ &- i\omega\alpha\left(-(u_{\mathrm{G}_1} - u_{\mathrm{A}})\sin\theta_0\cos\theta_0 + (w_{\mathrm{G}_1} - w_{\mathrm{A}})\cos^2\theta_0\right)\end{aligned} \tag{A 16}$$

$$-\omega^2 I_{1,\mathrm{G}_1}\theta = \left(\mathbf{M}_{E_1,\mathrm{G}_1} + \mathbf{M}_{R_1,\mathrm{G}_1} + \mathbf{M}_{H_1,\mathrm{G}_1}\right)\cdot\mathbf{y} + M_{L_{2\to1}} \tag{A 17}$$

$$\begin{aligned}-m_2\omega^2\left(u_{\mathrm{A}} + w_{\mathrm{G}_2 r}\theta\right) = &-\left(\mathbf{F}_{L_{2\to1}} \cdot \mathbf{x}''\right)\cos\theta_0 \\ &+ i\omega\alpha\left((u_{\mathrm{G}_1} - u_{\mathrm{A}})\sin^2\theta_0 - (w_{\mathrm{G}_1} - w_{\mathrm{A}})\cos\theta_0\sin\theta_0\right)\end{aligned} \tag{A 18}$$

$$\begin{aligned}-m_2\omega^2\left(w_{\mathrm{A}} - u_{\mathrm{G}_2 r}\theta\right) = &-\left(\mathbf{F}_{L_{2\to1}} \cdot \mathbf{x}''\right)\sin\theta_0 \\ &+ i\omega\alpha\left(-(u_{\mathrm{G}_1} - u_{\mathrm{A}})\sin\theta_0\cos\theta_0 + (w_{\mathrm{G}_1} - w_{\mathrm{A}})\cos^2\theta_0\right)\end{aligned} \tag{A 19}$$

$$-m_2\omega^2\left((u_{\mathrm{G}_2 r}^2 + w_{\mathrm{G}_2 r}^2)\theta - u_{\mathrm{G}_2 r}w_{\mathrm{A}} + w_{\mathrm{G}_2 r}u_{\mathrm{A}}\right) = -M_{L_{2\to1}} \tag{A 20}$$

where ω is the radiant frequency. With the equation corresponding to the geometric constrain (A 14) there is a total of seven equations for seven unknowns: u_{G_1}, w_{G_1}, u_{A}, w_{A}, θ, $\left(\mathbf{F}_{L_{2\to1}} \cdot \mathbf{x}''\right)$ and $M_{L_{2\to1}}$. The latter two unknowns do not correspond to body motions and are therefore not of direct interest. They can get rid of by combining the above equations as follows: (A 15)+(A 18), (A 16)+(A 19), (A 17)+(A 20) and (A 19)$\cos\theta_0$-(A 18)$\sin\theta_0$. With (A 14) this yields a system of five independent equations. To express the system in matrix form it is convenient to use the notation introduced in (2.14). The hydrodynamic loads $\mathcal{H}_1$, $\mathcal{R}_1$ and $\mathcal{E}_1$ applied to body 1 are given by:

$$\mathcal{H}_1 = \boldsymbol{C}\begin{pmatrix}\xi_1\\ \xi_2\\ \xi_3\end{pmatrix}, \mathcal{R}_1 = \left(-\omega^2\boldsymbol{A} + i\omega\boldsymbol{B}\right)\begin{pmatrix}\xi_1\\ \xi_2\\ \xi_3\end{pmatrix} \text{ and } \mathcal{E}_1 = \mathbf{E} \tag{A 21}$$

where $\boldsymbol{C}$, $\boldsymbol{A}$ and $\boldsymbol{B}$ are respectively the hydrostatic, added mass and hydrodynamic damping 3×3 matrices computed by WAMIT. $\mathbf{E}$ is wave exciting force 3×1 vector also computed by WAMIT. The final matrix equation of the system is given by (2.15).

B. Optimisation model

R summary of the model fitted to the $score_{50}$ data:

```
Call:
lm(formula = score50 ~ I(theta0^2) + I((log(alpha))^2) + theta0 +
    m2 + log(alpha) + theta0:m2 + theta0:log(alpha) + theta0:wG2r +
    m2:log(alpha) + m2:wG2r + log(alpha):wG2r)

Residuals:
      Min        1Q    Median        3Q       Max
-0.090720 -0.010297  0.003485  0.012606  0.049624

Coefficients:
                    Estimate Std. Error t value Pr(>|t|)
(Intercept)       -1.614e+00  1.039e-01 -15.543  < 2e-16 ***
I(theta0^2)       -1.641e-04  4.423e-06 -37.115  < 2e-16 ***
```

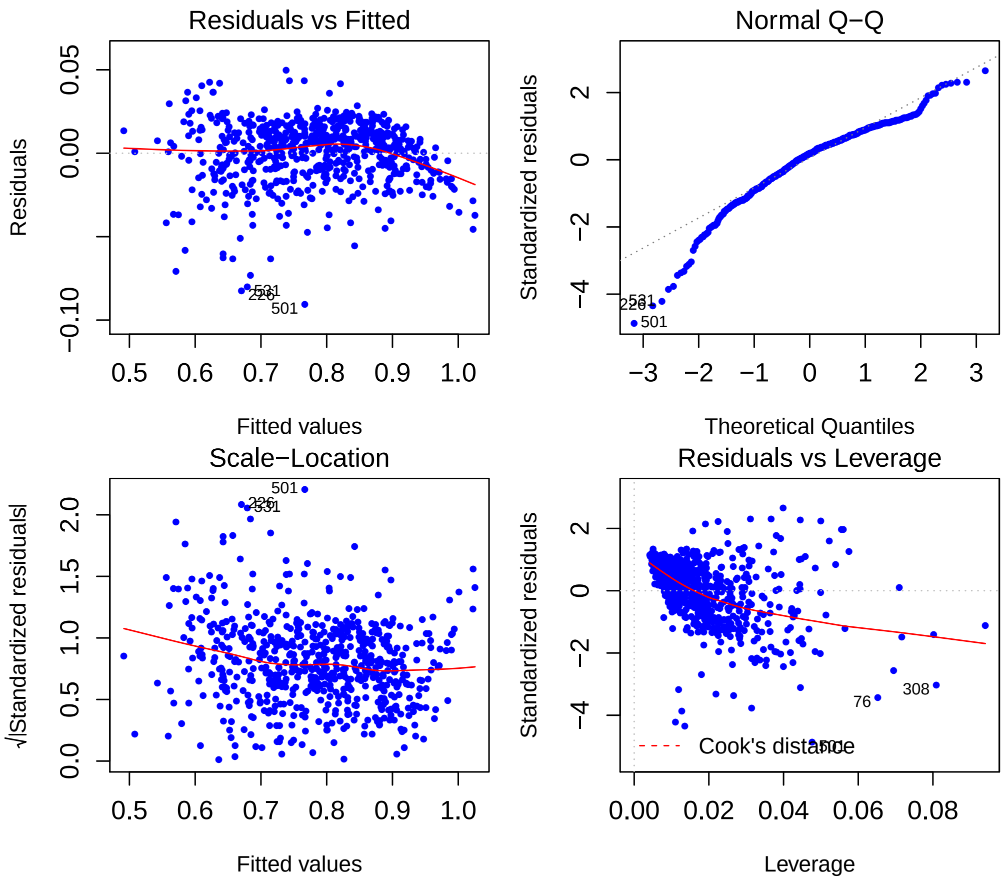

Figure 1. Test plots for the analytical model fitted to the $score_{50}$ data.

```
I((log(alpha))^2) -6.479e-02  4.282e-03 -15.130  < 2e-16 ***
theta0            -1.809e-02  7.689e-04 -23.531  < 2e-16 ***
m2                 2.134e-03  4.427e-04   4.821 1.80e-06 ***
log(alpha)         6.276e-01  4.069e-02  15.425  < 2e-16 ***
theta0:m2          2.093e-05  2.567e-06   8.156 1.90e-15 ***
theta0:log(alpha) -3.209e-04  1.589e-04  -2.019   0.0439 *
theta0:wG2r       -5.970e-03  1.048e-03  -5.699 1.86e-08 ***
m2:log(alpha)      4.651e-04  8.170e-05   5.692 1.93e-08 ***
m2:wG2r            2.867e-03  6.291e-04   4.557 6.25e-06 ***
log(alpha):wG2r   -2.419e-01  1.697e-02 -14.260  < 2e-16 ***
---
Signif. codes:  0 '***' 0.001 '**' 0.01 '*' 0.05 '.' 0.1 ' ' 1

Residual standard error: 0.01909 on 626 degrees of freedom
Multiple R-squared:  0.9674,Adjusted R-squared:  0.9668
F-statistic:  1688 on 11 and 626 DF,  p-value: < 2.2e-16
```

R summary of the model fitted to the $score_{40}$ data:

```
Call:
```

```
lm(formula = score40 ~ I(theta0^2) + I((log(alpha))^2) + theta0 +
    m2 + log(alpha) + theta0:m2 + theta0:log(alpha) + theta0:wG2r +
    m2:log(alpha) + m2:wG2r + log(alpha):wG2r)

Residuals:
      Min        1Q    Median        3Q       Max
-0.101843 -0.011747  0.004198  0.014489  0.055658

Coefficients:
                    Estimate Std. Error t value Pr(>|t|)
(Intercept)       -1.960e+00  1.191e-01 -16.449  < 2e-16 ***
I(theta0^2)       -1.780e-04  5.073e-06 -35.090  < 2e-16 ***
I((log(alpha))^2) -7.500e-02  4.912e-03 -15.268  < 2e-16 ***
theta0            -1.964e-02  8.821e-04 -22.270  < 2e-16 ***
m2                 2.286e-03  5.079e-04   4.500 8.08e-06 ***
log(alpha)         7.235e-01  4.668e-02  15.500  < 2e-16 ***
theta0:m2          2.435e-05  2.945e-06   8.271 7.99e-16 ***
theta0:log(alpha) -3.854e-04  1.823e-04  -2.114   0.0349 *
theta0:wG2r       -6.906e-03  1.202e-03  -5.747 1.42e-08 ***
m2:log(alpha)      5.475e-04  9.373e-05   5.841 8.32e-09 ***
m2:wG2r            3.644e-03  7.217e-04   5.048 5.85e-07 ***
log(alpha):wG2r   -2.880e-01  1.946e-02 -14.796  < 2e-16 ***
---
Signif. codes:  0 '***' 0.001 '**' 0.01 '*' 0.05 '.' 0.1 ' ' 1

Residual standard error: 0.0219 on 626 degrees of freedom
Multiple R-squared:  0.9645,Adjusted R-squared:  0.9638
F-statistic:  1545 on 11 and 626 DF,  p-value: < 2.2e-16
```

R summary of the model fitted to the $score_{20}$ data:

```
Call:
lm(formula = score20 ~ I(theta0^2) + I((log(alpha))^2) + (theta0 +
    m2 + log(alpha) + wG2r)^2)

Residuals:
      Min        1Q    Median        3Q       Max
-0.128440 -0.015711  0.004669  0.019928  0.067594

Coefficients:
                    Estimate Std. Error t value Pr(>|t|)
(Intercept)       -2.875e+00  1.681e-01 -17.100  < 2e-16 ***
I(theta0^2)       -2.092e-04  6.580e-06 -31.794  < 2e-16 ***
I((log(alpha))^2) -9.975e-02  6.432e-03 -15.509  < 2e-16 ***
theta0            -2.287e-02  1.129e-03 -20.255  < 2e-16 ***
m2                 2.631e-03  6.499e-04   4.049 5.78e-05 ***
log(alpha)         9.760e-01  6.272e-02  15.559  < 2e-16 ***
wG2r              -8.132e-01  2.571e-01  -3.163  0.00164 **
theta0:m2          3.200e-05  3.775e-06   8.475  < 2e-16 ***
theta0:log(alpha) -6.027e-04  2.349e-04  -2.566  0.01053 *
theta0:wG2r       -9.258e-03  1.545e-03  -5.993 3.47e-09 ***
m2:log(alpha)      7.059e-04  1.199e-04   5.887 6.43e-09 ***
m2:wG2r            4.712e-03  9.359e-04   5.035 6.27e-07 ***
```

rspa.royalsocietypublishing.org Proc R Soc A

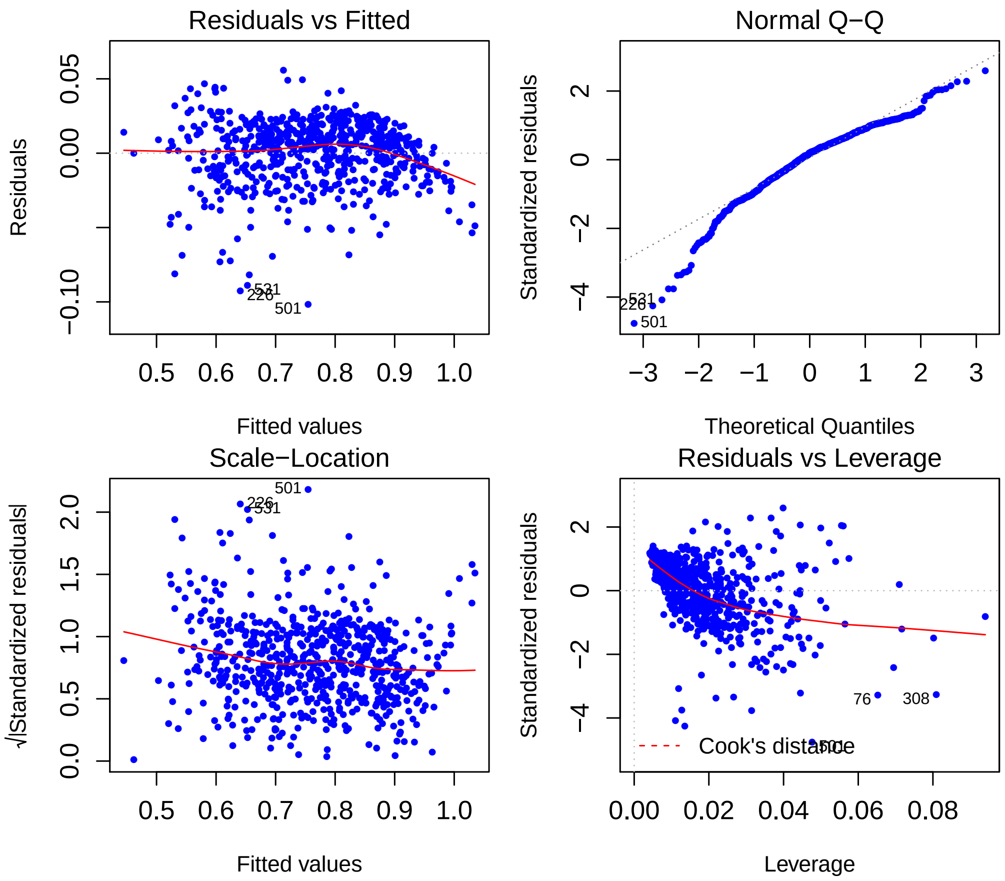

Figure 2. Test plots for the analytical model fitted to the $score_{40}$ data.

```
log(alpha):wG2r   -2.318e-01  5.306e-02  -4.369 1.46e-05 ***
---
Signif. codes:  0 '***' 0.001 '**' 0.01 '*' 0.05 '.' 0.1 ' ' 1

Residual standard error: 0.02802 on 625 degrees of freedom
Multiple R-squared:  0.9586,Adjusted R-squared:  0.9578
F-statistic:  1206 on 12 and 625 DF,  p-value: < 2.2e-16
```

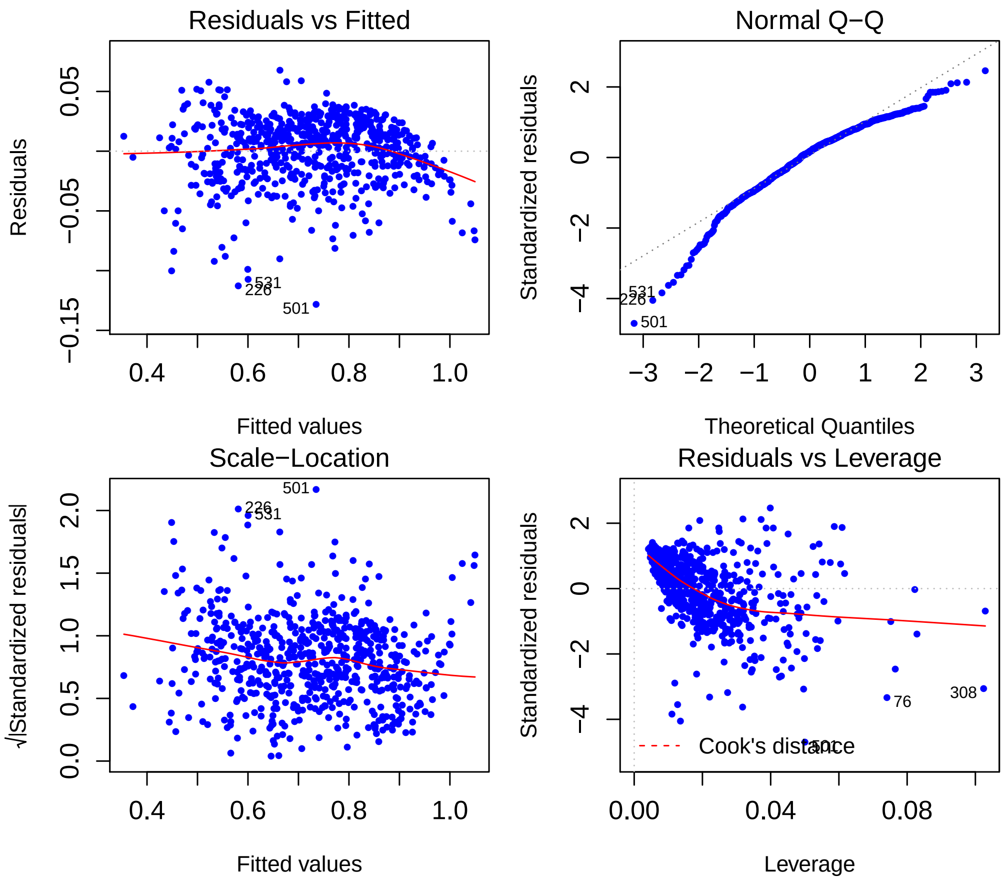

Figure 3. Test plots for the analytical model fitted to the $score_{20}$ data.
